# Proanthocyanidins and Flavan-3-ols in the Prevention and Treatment of Periodontitis—Immunomodulatory Effects, Animal and Clinical Studies

**DOI:** 10.3390/nu13010239

**Published:** 2021-01-15

**Authors:** Izabela Nawrot-Hadzik, Adam Matkowski, Paweł Kubasiewicz-Ross, Jakub Hadzik

**Affiliations:** 1 Department of Pharmaceutical Biology and Botany, Wroclaw Medical University, 50556 Wroclaw, Poland; izabela.nawrot-hadzik@umed.wroc.pl; 2 Department of Dental Surgery, Wroclaw Medical University, 50425 Wroclaw, Poland; pawel.kubasiewicz-ross@umed.wroc.pl (P.K.-R.); jakub.hadzik@umed.wroc.pl (J.H.)

**Keywords:** condensed tannins, proanthocyanidins, flavan-3-ols, periodontitis, gingivitis, gum disease, cranberry, *Camellia sinensis*, polyphenols, immunomodulatory, natural compounds, natural substances

## Abstract

This paper continues the systematic review on proanthocyanidins and flavan-3-ols in the prevention and treatment of periodontal disease and covers the immunomodulatory effects, and animal- and clinical studies, while the other part discussed the direct antibacterial properties. Inflammation as a major response of the periodontal tissues attacked by pathogenic microbes can significantly exacerbate the condition. However, the bidirectional activity of phytochemicals that simultaneously inhibit bacterial proliferation and proinflammatory signaling can provide a substantial alleviation of both cause and symptoms. The modulatory effects on various aspects of inflammatory and overall immune response are covered, including confirmed and postulated mechanisms of action, structure activity relationships and molecular targets. Further, the clinical relevance of flavan-3-ols and available outcomes from clinical studies is analyzed and discussed. Among the numerous natural sources of flavan-3-ols and proanthocyanidins the most promising are, similarly to antibacterial properties, constituents of various foods, such as fruits of *Vaccinium* species, tea leaves, grape seeds, and tannin-rich medicinal herbs. Despite a vast amount of in vitro and cell-based evidence of immunomodulatory there are still only a few animal and clinical studies. Most of the reports, regardless of the used model, indicated the efficiency of these phytochemicals from cranberries and other *Vaccinium* species and tea extracts (green or black). Other sources such as grape seeds and traditional medicinal plants, were seldom. In conclusion, the potential of flavan-3-ols and their derivatives in prevention and alleviation of periodontal disease is remarkable but clinical evidence is urgently needed for issuing credible dietary recommendation and complementary treatments.

## 1. Introduction

In the previous paper, we demonstrated that both free flavan-3-ols and oligomeric proanthocyanidins are very promising constituents for combating various bacteria involved in periodontitis pathogenesis [1]. Here, using the same systematic approach, we have selected and discussed the recent data on anti-inflammatory and immunomodulating activities of these compounds, including in vivo models and clinical studies.

**Figure 1:**
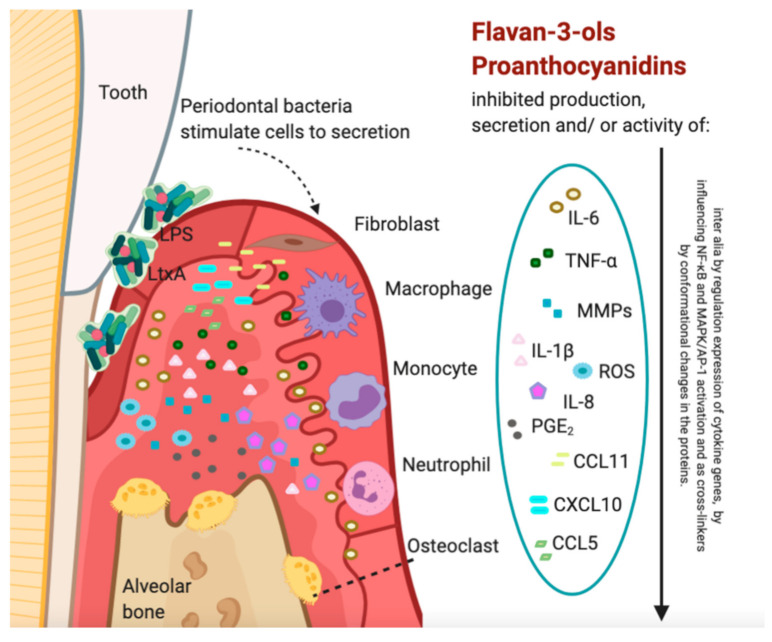
Schematic picture illustrating immunomodulatory activities of flavan-3-ols and proanthocyanidins in periodontitis. Some of the more important cytokines are shown in the figure. The figure was created using BioRender.com. Abbreviations shown in the figure: IL, interleukin; TNF-α, tumor necrosis factor α; MMP, matrix metalloproteinases; ROS, reactive oxygen species; PGE_2_, prostaglandin E_2_; CCL, C-C motif chemokine ligand; CXCL, C-X-C motif chemokine ligand; LPS, lipopolysaccharide; LtxA, Leukotoxin released by *A. actinomycetemcomitans*.

According to the latest concept of periodontitis etiopathology, the development of the disease requires the co-existence of dental plaque that accumulates on the teeth surface and the patient’s immune-inflammatory response [2]. Periodontal bacteria cause the mobilization of innate immune response (e.g., large phagocytes like macrophages, antigen-presenting dendritic cells (DCs), natural killer (NK) cells and neutrocytes) as well adaptive immunity mechanisms (T cells and B cells) that leads to the release of pro-inflammatory cytokines including interferon-gamma (IFNγ), interleukin-17 (IL-17), tumor necrosis factor-alpha (TNF-α), interleukin-1 and 6 (IL-1 and IL-6) and enzymes including in particular collagenases like matrix metalloproteinases (MMPs) [3] (Figure 1). By the inflammatory response the body protects itself against bacteria and their invasion inside the deeper tissues (such as bone). However, if the inflammatory process persists and is poorly regulated by the host, it can cause the most troublesome detrimental changes in periodontium tissue form and function such as periodontal pockets, attachment loss, gingival recessions, tooth mobility, tooth migration, and tooth loss [4]. Literature provides the data about anti-inflammatory and antioxidant effects of polyphenols which include flavan-3-ols and proanthocyanidins, drawing conclusions that they can be beneficial in the prevention and can be useful as a disease-controlling factor of a series of chronic diseases including diabetes, obesity, neurodegeneration, cancers, and cardiovascular diseases [5]. Our review focused on the activity the highly potent compounds among polyphenols in relation to periodontitis—a disease closely related to the inflammatory diseases mentioned above [2].

## 2. Methods

Search strategy, as well as inclusion, exclusion criteria, and data organization are described in our previous review [1], in which the antimicrobial activity of proanthocyanidins and flavan-3-ols in the prevention and treatment of periodontitis is discussed. In brief, a systematic review in compliance to PRISMA guidelines was performed. An electronic database search was conducted using PubMed, Web of Science and Scopus (accessed 23 December 2020).

**Figure 2:**
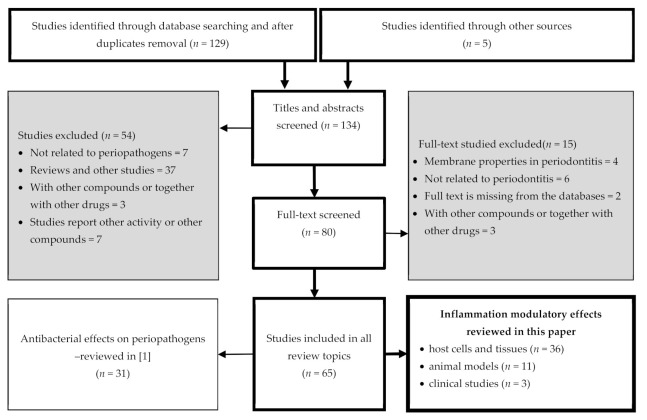
Flowchart of the article search strategy, exclusion criteria, study selection, and data management process. Of all 65 considered references, 50 are reviewed in this paper and 31 in [1], of which 16 references are included in both reviews.

The search terms included all combinations of the following key words: ‘periodontitis’ OR ‘periodontal diseases’ OR ‘gingivitis’ OR ‘gingival diseases’ AND ‘proanthocyanidins’ OR ‘condensed tannins’ OR ‘flavan-3-ols’ OR ‘catechin’ OR ‘epicatechin’ AND ‘anti-bacterial’ OR ‘antiadhesive’ OR ‘anti-inflammatory’, respectively. All titles with abstracts were imported into a citation manager program “Mendeley” (Elsevier, London, UK), and all duplicates were removed. References of imported articles were also screened for other relevant studies. Two investigators (N.-H.I. and K.-R.P.) independently reviewed the titles and abstracts of the imported references to determine whether they met the inclusion and exclusion criteria (Figure 2).

Finally, 31 studies were reviewed in [1] (antibacterial effects) and 50 studies are included in the present paper (Figure 2).

## 3. Immunomodulatory Effects of Proanthocyanidins or Flavan-3-ols on Host Cells and Tissues, In Vitro Studies

### 3.1. Influence on Matrix Metalloproteinases (MMPs)

It was already proved that matrix metalloproteinases (MMPs) play a very important role in the process of periodontal connective tissue destruction. MMPs are a calcium-dependent zinc-containing endopeptidases, responsible for the tissue remodeling and degradation of the extracellular matrix (ECM), including collagens, elastin, gelatin, matrix glycoproteins, and proteoglycan [6]. Major cell types that can be found in the periodontium, like fibroblasts, neutrophils, and macrophages, release these proteolytic enzymes [7]. These proteolytic enzymes are secreted as latent proenzymes (except membrane type (MT)-MMPs) and they must be later activated by tissue, plasma or bacterial proteinases extracellularly or at the cell surface. Under normal condition, MMPs play an important role in the healing of the wounds, the process of angiogenesis, and remodeling of the gingival tissue [8]. However, in periodontitis host cells are threatened by the periopathogens and their products such lipopolysaccharides (LPS) of Gram-negative bacteria. As a result, an increased production of MMPs can be observed that influences the degradation of periodontal ligaments, the loss of gingival collagen, and the resorption of alveolar bone, leading to destruction of periodontal tissues [9]. Increased activity of the enzymes-collagenases (matrix metalloproteinases 1 and 8) and gelatinases (matrix metalloproteinases 2 and 9) have been described in in the gingival crevicular fluid and in the inflamed gingival tissues of patients with periodontitis [10]. MMPs have the ability to activate and inactivate or even antagonize the biological functions of cytokines and chemokines. Through the influence on the cytokines and chemokines MMPs can regulate the inflammatory process by promoting or suppressing it. On the other hand, when the inflammatory cells are stimulated by cytokines and chemokines a production of MMPs may be induced [11]. Many studies proved that proanthocyanidins could inhibit activity of matrix metalloproteinases (Table 1).

Particularly, the *n*-butanol fraction from the *Ulmus macrocarpa* Hance bark, defined as elm extract (contain 20% of procyanidins) and the mixture of procyanidin oligomers (composed of 3 to 12 flavan-3-ol monomers, with average molecular weight of 1518) isolated from elm extract in range 100–1000 μg/mL exhibited inhibitory effects on the MMPs, present in gingival crevicular fluid (GCF) of adult patients with periodontal disease (mainly, MMP-8 and MMP-9) and on the pro and active forms of MMP-2 (from the conditioned media of cultured periodontal ligament (PDL) cells treated with a periodontopathogen, *Treponema lecithinolyticum*) [10]. The inhibition of enzyme activity by procyanidin oligomers was more effective than by the elm extract, with IC50 values 25 and 33 µg/mL for GCF collagenases (mostly MMP-8 and MMP-9) and MMP-2, respectively. Moreover, elm extract and procyanidin oligomers inhibited proteolytic enzymes of two periopathogens, *T. denticola,* and *P. gingivalis* responsible for degradation of the interstitial and basement membrane collagens as well as activating of matrix metalloproteinases e.g., MMP-8, MMP-9 or MMP-1, MMP-3, and MMP-9 [10].

Free (non-polymerized) galloylated flavan-3-ols such as (−)-epicatechin gallate and (−)-epigallocatechin gallate (EGCG) from green tea also inhibited collagenase activity, achieved total inhibition at 50 µg/mL [46]. Other tested compounds, without the gallate residue such as catechin, epicatechin, gallocatechin, and epigallocatechin had no effect on collagenase. Makimura reported also that ethyl acetate fraction from tea leves (*Camellia sinensis*)*,* which contained six above catechins inhibited collagenolytic proteases in gingival crevicular fluids (GCF) and in culture supernatants of *Porphyromonas gingivalis* [46]. Similar results were observed by Demeule et al. [45]. Green tea polyphenols, especially (−)-epigallocatechin gallate (EGCG) and (−)-epicatechin gallate (ECG), inhibited matrix metalloproteinase (MMP)-2, MMP-9, MMP-12 and proMMP-2 activities in the range of micromolar concentrations. In the following years, more studies confirmed the inhibitory effects of proanthocyanidins and its galloylated monomers on production and/or activity of matrix metalloproteinases MMPs (MMP-1, MMP-2, MMP-3, MMP-7, MMP-8, MMP-9, and MMP-13) involved in periodontitis (Table 1). Among them were proanthocyanidins and gallate catechins from green tea [21,44], theaflavins from black tea [20], A-type cranberry proanthocyanidins (AC-PAs) isolated from cranberries (*Vaccinium macrocarpon* fruits) [28,31,32,38,41], proanthocyanidins from blueberries of two North American species—*Vaccinium corymbosum* [19], and *V. angustifolium* [22].

In opposition to the above results are results obtained by Lombarto et al. who showed [23] that AC-PAs and EGCG, individually or in combination, had no effect on the regulation of MMP (1, 2, 3, 7, 8, 9, 10, 12, and 13) and tissue inhibitors of metalloproteinases - TIMP (1, 2, 3, and 4) secretion but inhibited the secretion of several cytokines in the (3D) co-culture model of gingival epithelial cells and fibroblasts stimulated with *A. actinomycetemcomitans* LPS (Table 1).

### 3.2. Influence on Bone Tissue Resorption

Yun et al. [44] reported an inhibitory effect of EGCG (20 µM) on the MMP-9 gene expression in osteoblasts and on the formation of osteoclasts, which suggested that EGCG may prevent the alveolar bone resorption that occurs in periodontal diseases leading to teeth loss. Importantly, in the periodontal disease an enhanced osteoclastogenesis can occur due to the presence of the of inflammatory cytokines that stimulates osteoclast proliferation or promotes the differentiation of progenitor cells. Mature osteoclasts that derive from hematopoietic monocyte/macrophage precursors under the action of RANKL (receptor activator of NF-κB ligand) and M-CSF (macrophage colony-stimulating factor) mediate the destruction of the alveolar bone by attaching to the bone surface and promoting mineral dissolution. The demineralized bone matrix is later degraded by proteases such as cathepsin K and metalloproteinases (MMPs) [32]. Tanabe et al. [32] showed that AC-PAs have influence the osteoclast formation and bone resorption activity. In a range of 10–100 μg/mL, AC-PAs inhibited RANKL-dependent osteoclast differentiation, as well as secretion of both MMP-2 and MMP-9 but secretion of IL-8 was increased. IL-8 from normal human bone marrow stromal cells inhibits the bone resorbing activity of osteoclasts [47].

**Figure 3:**
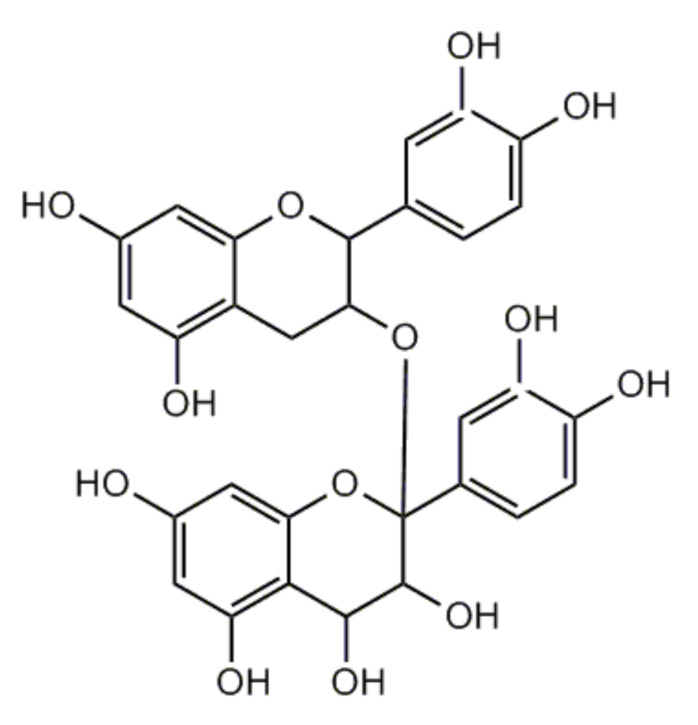
Structure of untypical proanthocyanidins isolated from fruits of *Vitis vinifera* used in the study of Huang et al. [13].

Huang et al. [13] reported that proanthocyanidins (PA) may have an influence on the bone regeneration in the host inflammatory microenvironment by suppressing NF-κB signaling pathway and therefore may be a potential inducer of bone regeneration. In this study an effect of proanthocyanidins on osteogenic differentiation of human periodontal ligament fibroblasts (PDLFs) with or without TNF-α stimulation was tested and the biological mechanism was explored. The assumption was that periodontal ligament fibroblasts are capable of differentiating into osteoblasts, but pro-inflammatory cytokines like TNF-α inhibit this process. Osteogenic differentiation and mineralization associated markers were detected by qRT-PCR, alizarin red S staining, and alkaline phosphatase (ALP) activity assay. In result, proanthocyanidins in low concentration (0.1 μg/mL, 1 μg/mL, 10 μg/mL) significantly upregulated expression of osteogenesis-related genes and proteins and ALP activity in PDLFs compared with the control. However, proanthocyanidins at higher concentrations of 30 μg/mL and 50 μg/mL significantly suppressed the alkaline phosphatase activity of PDLFs. For the rest assay, authors used only lower concentration of PA (0.1 μg/mL, 1 μg/mL, 10 μg/mL). Proanthocyanidins in concentration of 1 μg/mL significantly reversed inhibition of osteogenesis related gene and protein expression, alkaline phosphatase activity, and mineralization caused by TNF-α. The authors also suggested that proanthocyanidins may reverse TNF-α inhibited osteogenic differentiation via NF-κB signaling pathway. These authors used commercial proanthocyanidins claimed to possess an untypical for proanthocyanidins structure (Figure 3) and with molecular weight = 594.52. The supplier’s website states that proanthocyanidins have been isolated from grapes (the fruits of *Vitis vinifera* L.).

### 3.3. Influence on Cytokines

The overproduction and secretion of inflammatory cytokines by resident and immune cells modulate the progression and severity of periodontitis. Increase of such proinflammatory cytokines as: IL-1α, IL-1β, TNF-α, IL-6, and IL-17 were shown in patients with acute or chronic periodontitis [48]. More specifically, TNFα can be found at high levels in gingival crevicular fluid (GCF) and in diseased periodontal tissues, where it is positively correlated with MMPs and RANKL expression. Human and animal studies confirmed that TNF-α plays a central role in inflammatory reaction, alveolar bone resorption, and the loss of connective tissue attachment. Moreover, TNF-α up-regulates the production of other proinflammatory innate immunity cytokines, such as IL-1β and IL-6 associated with inflammatory cell migration and osteoclastogenesis [48]. IL-1β plays an important role in the pathogenesis of periodontitis also by regulation of the IL-6 production in a variety of cell types, including fibroblasts and epithelial cells [26]. Similar to bacterial LPS, cellular response to cytokines or chemokines (e.g., IL-1β) can be mediated via signaling cascades, including NF-κB and MAPK/AP-1 pathways, which lead to gene expression of certain proteins (for example IL-6). There are more and more studies proving that proanthocyanidins and flavan-3-ols inhibit the secretion of cytokines by influencing NF-κB and MAPK/AP-1 activation (Table 1) [13,14,18,19,20,22,26,28,38].

Many studies have shown inhibition of production and/or secretion of inflammatory cytokines by proanthocyanidins. Bodet et al. [42] demonstrated that proanthocyanidin-enriched cranberry fraction at concentrations 25–50 µg/mL, significantly inhibited the IL-6, IL-8, and PGE_2_ production by gingival fibroblasts stimulated with the *Aggregatibacter actinomycetemcomitans* lipopolysaccharide (LPS). The most spectacular inhibitory effect was seen towards IL-8 that belongs to chemokines (CXCL8) also known as neutrophil chemotactic factor. It directs the migration of polymorphonuclear leukocytes, monocytes, and macrophages to the infection site. Increased level of IL-8 was observed in the gingival crevicular fluid of inflamed periodontal sites [42]. PGE_2_ is another proinflammatory molecule involved in destructive process in periodontal disease. It is secreted in response to pro-inflammatory cytokines, periodontopathogens and LPS. The cranberry fraction significantly inhibited PGE2-response even at low tested concentration—25 μg/mL, and reduced COX-2 protein expression—the enzyme involved in PGE2 production. Moreover, cranberry fraction influence on the phosphorylation and expression of various intracellular proteins (Jun, Fos, MKK3, MKK6, Rac1, and Mnk1) which are implicated in cytokine production. Bodet et al. concluded that cranberry fraction may act especially via a downregulation of AP-1 activity [42]. Feldman and Grenier [31] showed an inhibitory effect of 25 or 50 µg/mL of A-type cranberry proanthocyanidins (APA) on TNF-α, IL-6, and IL-8 secretion in a macrophage model. The 50 µg/mL concentration of APA reduced the LPS-induced secretion of TNF-α, IL-6 and IL-8, by about 50%, but had not influence on IL-1β. A significant reduction in IL-1β secretion was seen when ACPA was used together with licochalcone A (chalcone, not proanthocyanidin). Further studies on proanthocyanidins, in the predominant amount on A-type cranberry proanthocyanidins, prove their influence on the secretion and production of interleukins, as well as provided explanation of molecular mechanisms responsible for this activity [12,17,23,26,27,28,29,37] (Table 1).

Galarraga-Vinueza et al. [12] revealed that cranberry concentrate from capsules (Uriach-Aquilea OTC, Barcelona, Spain) containing 130 mg A-type PAs significantly decreased M1 polarization and increased M2 polarization in LPS-stimulated macrophages. M1 phenotype of macrophage are activated by bacteria sub-products like lipopolysaccharide and are associated with the secretion of pro-inflammatory cytokines (such as IL-1β, IL-6, IL-8), whereas a M2 phenotype of macrophages are activated by alternative ways and are associated with the secretion of anti-inflammatory cytokines (such as IL-10) and growth factors which enhance tissue regeneration. Galarraga-Vinueza et al. [12] confirmed the effect of A-type PAs (50 and 100 µg/mL) on cytokine expression-proinflammatory cytokines: IL-8 and IL-6 were significantly downregulated in LPS-stimulated macrophages and A-type PAs, whereas an anti-inflammatory IL-10 was upregulated. No influence on expression of IL-1ß was seen. Lagha et al. [17] showed that fraction of proanthocyanidins (PAs) from cranberries at a concentration of 15.625–125 µg/mL markedly reduced cytotoxicity of leukotoxin on macrophages and significantly reduced (by about 80–90% at 15.625 and more than 98% at 125 µg/mL) release of caspase-1, IL-1β, and IL-18 from LtxA-induced macrophages. Leukotoxin (LtxA), released by *A. actinomycetemcomitans* is an important virulence factor playing a critical role in the pathogenic process of localized aggressive periodontitis (LAP). LtxA affects immune cells by activates pyroptosis of monocytes and macrophages and inducing the release of pro-inflammatory cytokines. Pyroptosis is the inflammatory form of programmed cell death, involves the activation of caspase-1, which in turn coverts of pro-IL-1β and pro-IL-18 to the biologically active forms. In macrophages, pyroptosis leads to the formation of pores in the plasma membrane which allows secretion of IL-1β and IL-18, cytokines known as damage-associated molecular patterns (DAMPs) and contribute to the progression of periodontitis by increasing cell migration and osteoclastogenesis [49,50]. Moreover, PAs reduced the expression of CIAS and P2X7 genes (increase by LtxA, in macrophages) by about 30–45%, similarly for a range 15.625–125 µg/mL [17]. This is important because the P2X7 receptor activation and CIAS activation leads to the rapid formation of membrane pores and to the release of IL-1β and IL-18. Moreover, the cranberry proanthocyanidins blocked the binding of LtxA to macrophages and reduced ROS and superoxide production in LtxA-induced macrophages.

Cranberry proanthocyanidins (PAs) can differently affect interleukins secretion/production, depending on a cell type. In lipopolysaccharide-stimulated normal human gingival fibroblast, cranberry non-dialyzable material (NDM) rich in proanthocyanidins decreased level of IL-6, what is consistent with other studies, but NDM significantly increased IL-6 in lipopolysaccharide-stimulated human gingival fibroblast cells from a patient suffering from aggressive form periodontitis (AgP fibroblasts) [28]. This increasing level of IL-6 occurred only in the presence of LPS; NDM alone did not show a significant increase in production of IL-6. Simultaneously, NDM inhibited NF-κB activity (increased by LPS treatment) in AgP fibroblasts hinting at the involvement of other molecular mechanisms of IL-6 regulation in these cells.

Influence of proanthocyanidins and flavan-3-ols from other source than cranberries on the secretion and production of interleukins was also demonstrated in several studies (Table 1). Jekabsone et al. [15] reported that *Pelargonium sidoides* root extract (PSRE) and especially proanthocyanidin fraction from PSRE (PACN) exhibits a strong, antibacterial properties (against *Aggregatibacter actinomycetemcomitans)*, anti-inflammatory and gingival tissue protecting properties under periodontitis-mimicking conditions. The cells (gingival fibroblast, bone marrow-derived macrophages (BMDM) or human peripheral blood mononuclear cells (PBMCs) were stimulated using lipopolysaccharide (and IFNγ for BMDM) and treated with 50 µg/mL and 100 µg/mL of PSRE or PACN. The extracts protected human gingival fibroblast from *A. actinomycetemcomitans* infection, decreased lipopolysaccharide-induced release of IL-8 and prostaglandin E2 from gingival fibroblasts and IL-6 from leukocytes, blocked expression IL-1β, iNOS and COX-2 but not TNF-α. Stronger anti-inflammatory activity of proanthocyanidin fraction (PACN) than root extract (PSRE) was associated with higher amounts of prodelphinidins. The study also reported that PSRE and PACN (100 µg/mL) blocked the surface presentation of CD80 and CD86 (surface markers of proinflammatory M1 phenotype) in LPS + IFNγ-treated macrophages, whereas PACN was characterized by stronger activity. These results indicate that both PACN and PSRE are potent in preventing macrophage conversion to proinflammatory M1 phenotype under exposure to LPS.

Low concentrations (7.9–62.5 μg/mL) extracts from a black and green tea as well as their flavan-3-ols (epigallocatechin-3-gallate, theaflavins) have influence on production and secretion proinflammatory cytokines. They reduce the epithelial gingival barrier dysfunction caused by TNF-α and modulate the hosts inflammatory response. They inhibited the activation of NF-κB and caspase-1 as well as reduced IL-1β secretion by macrophages (at 62.5 μg/mL by more than 94%, except black tea—64.5%), and secretion IL-8 (only black tea required higher concentration than 62.5 μg/mL for more than 70% inhibition) by human oral epithelial cells stimulated with recombinant TNF-α [16]. The green tea extract showed higher activity than black tea extract. Other studies have confirmed the inhibitory effect of tea derived flavan-3-ols on the secretion of pro-inflammatory cytokines from LPS stimulated macrophages as well from cytokines-stimulated gingival cells (Table 1) [14,23,24,36]. Ben Lagha et al. [20] presented consistent results in which they proved inhibitory effect theaflavins (TFs) from black tea on the secretion of pro-inflammatory cytokines from *Porphyromonas gingivalis* treated macrophages and on the activation of the NF-κB signaling pathway (Table 1). Lombardo Bedran et al. [24,25] in studies on green and black tea and their main galloylated flavan-3-ols revealed the ability of these compounds to induce human beta-defensin (hBD) secretion in gingival epithelial cells. HBDs are antimicrobial peptides secreted by gingival epithelium in response to periopathogens. HBDs interact with the bacterial cell membrane, and lead to pore formation and finally to the lysis of major periopathogens. Evidence indicated that level of hBDs is higher in healthy gingival tissues than in periodontal gingival tissues and that some periopathogens like *P. gingivalis*, are capable to down-regulate hBD expression by epithelial cells and/or to inactivate hBDs by the means of the proteolytic cleavage [25]. Both green and black teas and their galloylated flavan-3-ols stimulated secretion of hBDs and increased expression of the *hBD* gene in gingival epithelial cells as well as prevented the degradation of hBD1 and hBD2 by periopatogen *P. gingivalis.* Again, the tested non-galloylated flavan-3-ols-theaflavins failed to induce secretion of significant amounts of hBDs by the epithelial cells.

In addition to the inhibitory effect of EGCG on innate immune response (e.g., IL-1, IL-6, TNF-α), an influence on adaptive immunity mechanisms (Th1, Th2, Th17, and Tregs) was demonstrated. Hosokawa et al. [30] showed influence of EGCG on Th2-type chemokines, such as CCL11 production. The EGCG in range 3.125–50 µg/mL decreased CCL11 production in IL-1β/IL-4 or TNF-α/IL-4-stimulated human gingival fibroblasts (HGFs) in a concentration dependent manner (almost total reduction at 50 µg/mL). Moreover, they demonstrated that ERK and JNK activations, related to CCL11 production in HGFs, are inhibited by EGCG treatment. The same group demonstrated an inhibitory effect of EGCG and ECG on CXC chemokine ligand 10 (CXCL10 production (about 60% inhibition by 50 µg/mL) in human gingival fibroblasts (HGFs) stimulated oncostatin M (OSM)—cytokine belonging to the interleukin family [35]. CXCL10 is a Th1-type chemokine which plays a key role in the recruitment of Th1 cells, and thus in the development of periodontal disease. It is supposed that EGCG and ECG suppressed production of CXCL10 through the inhibition of phosphorylation of signal transduction molecules like JNK, Akt and STAT3 phosphorylation as well as by suppressed OSMRβ expression in stimulated HGFs [35]. Influence on adaptive immunity mechanisms was also proved for cranberry AC-PAs [37]. AC-PAs significantly decreased the secretion of C-C motif ligand 5 (CCL5) from *P. gingivalis*-stimulated oral epithelial cells (100 µg/mL of AC-PAs reduced secretion of CCL5 and also IL-8 by more than 80%). The CCL5 chemokine (has significant chemotactic activity for Th1 cells as well basophiles, eosinophiles, monocytes [37].

In addition to the above, there is a couple of other well studied sources of proanthocyanidins with proven anti-inflammatory activities linked with periodontitis.

*Castanopsis lamontii* water extract (CLE), (400 μg/mL) rich in epicatechin (EC) and procyanidin B2 (PB2) as well as EC (120 µg/mL) and PB2 (34.4 µg/mL) alone, has suppressed in a significant manner the lipopolysaccharide-stimulated inflammation by inactivating the TLR-4/NF-κB/iNOS and TLR-4/NF-κB/COX-2 pathways [18]. All tested samples (CLE, EC, PB2) decreased the release of NO, PGE2, and TNF-α from stimulated-LPS mouse macrophage RAW264.7. PB2 appeared to be much more potent in suppressing the lipopolysaccharide-stimulated inflammatory response than EC.

Ben Lagha et al. [19] proved inhibitory effect of proanthocyanidins isolated from highbush blueberry (*Vaccinium corymbosum*) on the secretion of pro-inflammatory cytokines from LPS-*Aggregatibacter actinomycetemcomitans* treated macrophages and on the activation of the NF-κB signaling pathway. PAs at 125 µg/mL reduced the secretion of IL-1β, TNF-α, IL-6, and CXCL8 by 75.34%, 81.64%, 48.27%, and 90.19%, respectively [19]. Similarly, promising results were reported in the study on ethanolic lowbush blueberry extract (*Vaccinium angustifolium)* [22]. A pre-treatment of macrophages with the blueberry extract (in concentration of 62.5 μg/mL) and then stimulation with *Fusobacterium nucleatum* inhibited the secretion of IL-1β, TNF-α, and IL-6 by 87.3%, 80.7%, and 28.2%, respectively. The secretion of the chemokine CXCL8 was affected by 500 μg/mL, 250 μg/mL, or 125μg/mL extract, decreased CXCL8 secretion by 79%, 57.9%, and 11.2% respectively [22].

Proanthocanydin- enriched extract from *Myrothamnus flabellifolia,* plants traditionally used for treatment of gingival inflammation and periodontitis in South Africa, decreased gene expression of IL-1β, IL-8 and TNF-α, and level of IL-6 in KB cells, pre-incubated with MF (10 µg/mL and 100 µg/mL) and infected with *Porphyromonas gingivalis* [33].

### 3.4. Influence on Reactive Oxygen Species (ROS)

ROS and reactive nitrogen species (RNS) production by immune cells stimulated by periopathogens is an important factor in pathogenesis of periodontitis [43]. Their overproduction can lead to oxidative damage to healthy gingival tissue, periodontal ligament, and alveolar bone. Study of Houde et al. [43] showed that the stimulation of macrophages with lipopolysaccharide from *Aggregatibacter. actinomycetemcomitans* and *Fusobacterium nucleatum* induces increased NO and ROS release. However, macrophages pretreated with non-cytotoxic concentrations of grape seed extract (GSE) containing 95% oligomeric proanthocyanidins significantly inhibited free radical generation by inhibiting the production of the proinflammatory mediators NO and ROS and by modulating iNOS protein expression. Significant decrease of ROS (e.g., superoxide) production by macrophages (exposed to LtxA) was also observed in the presence of cranberry PAs [17]. The addition of 125 µg/mL of PAs reduced ROS and superoxide production by 92.2% and 72.7%, respectively. These outcomes are supported by the results from animal studies [51], [52], further discussed below.

## 4. In Vivo Studies Reporting Influence Proanthocyanidins or Flavan-3-ols on Periodontitis in Animal Models

Among the reviewed studies, eleven reported the influence of proanthocyanidins or flavan-3-ols on periodontitis in animal models. Three of them used grape seed proanthocyanidin extract [52,53,54]; four used the flavan-3-ols [14,39,55,56]; two used green tea extract [57,58]; finally, one study used cranberry (*Vaccinium macrocarpon*) juice [29] and one unverified commercial proanthocyanidin (PA) [51]. Toker et al. [53] presented results which indicated that grape seed proanthocyanidin extract (GSPE) can substantially decrease periodontal tissue inflammatory process and alveolar bone loss by decreasing MMP-8 and hypoxia-inducible factor 1-alpha (HIF-1-α) levels and increase osteoblast activity in diabetic rats with experimentally induced periodontal disease (Table 2). Giving 100 mg/kg and 200 mg/kg doses of grape seed proanthocyanidin extract (GSPE) administered by oral gavage to rats with induced diabetes and periodontitis significantly decreased alveolar bone loss, inflammatory cell numbers, MMP-8 and HIF-1-α levels compared to rats with diabetes + periodontitis but without GSPE. Moreover, the osteoblast number increased significantly in the GSPE groups compared to the periodontitis and diabetes + periodontitis groups. Oral administration of commercial grape seed proanthocyanidins (PC) [52] to rats with experimentally induced periodontitis (EP) revealed that PC enhanced the host resistance and inhibited the oxidative stress. In serum, proanthocyanidins (PC) significantly decreased reactive oxygen species, lipid peroxides, lysosomal enzymes, acute phase proteins and they increased antioxidant levels. Histopathological evidence of experimentally induced periodontitis without PC showed cellular infiltration of inflammatory cells whereas the groups treated with proanthocyanidins demonstrated only scattered inflammatory cells. Similar anti-inflammatory effect of grape seed extract (GSE) was observed in Ozden et al. study [54].

Cai et al. [55] study indicated that EGCG alleviates *P. gingivalis* induced periodontitis in the animal model. The mice orally inoculated with *P. gingivalis* in PBS, received sterile food and drunk water with 0.02% epigallocatechin-3-gallate solution in the period from 8 weeks to 15 weeks. It was found that epigallocatechin-3-gallate has significantly reduced alveolar bone resorption as well as decreased the high expressions (caused by *P. gingivalis* infection) of inflammatory cytokines and other mediators both in serum and in gingival tissue (details in the Table 2) what is consistent with a previous study of Lee y et al. [39], in which epigallocatechin-3-gallate showed a suppressing effect one the progression of periodontitis, by diminishing Cyr61 expression (a potential osteolytic mediator) in osteoblast cells and, subsequently, macrophage chemotaxis into the lesions. Cho et al. [56] observed decreased IL-6 and TNF expression in the tissue of rats orally fed EGCG compared to the group without EGCG. Downregulation of TNF-α and IL-6 expression by EGCG led to a decrease of the number of osteoclast number as well as decrease in their activity, which finally has resulted lower bone loss. They also noticed reduced collagen destruction in EGCG group. Similar results achieved Lee at al. [14] studying catechin, another of major flavan-3-ols in green tea. They showed that catechin has reduced the level of bone loss in mouse animal model with a *P. gingivalis* induced periodontitis.

In turn, Polak et al. [29] showed that cranberry non-dialyzable material (NDM) consumption by mice infected by *P. gingivalis* and *F. nucleatum* has lowered the alveolar bone loss compared to the mice with infection but without NDM treatment. Moreover, in subcutaneous chamber model of inflammation, NDM alone has been shown to reduce TNF-α levels induced by the mixed infection. In vivo studies were supported by in vitro study (Table 1). 

## 5. Clinical Studies

Until this moment, only three studies have been published pertaining to the use of proanthocyanidins or flavan-3-ols in periodontal disease in humans [59,60,61]. Two of them relate to the use of local delivery drug therapy with green tea extracts. Specifically, a thermo-reversible sustained-release system with incorporated green tea extract and hydroxypropylcellulose strips containing green tea catechin were used. Local therapeutic systems turned out to be effective in reducing periodontal pockets and inflammation [60,61] (details in Table 3). However, the weakness of Hirsawa et al. study [61] was the limited number of subjects in experimental group, as only 6 patients were treated. Díaz Sánchez et al. [59] were the only one to design study using pills rich in oligomeric proanthocyanidins. In this clinical study, 10 of 20 healthy volunteers with an induced gingivitis took the experimental treatment with oligomeric proanthocyanidins supplement administered orally as a dissoluble pill. According to Diaz Sánchez et al., the supplement caused improvement in the periodontal tissues condition during the period of treatment [59]. Although this study does not refer to periodontitis but to reversible gingivitis, the positive effect of the use oligomeric proanthocyanidins draws attention and encourages further clinical research.

## 6. Conclusions

Among the numerous in vitro studies (36) on the immunomodulatory effect of proanthocyanidins or flavan-3-ols on the host cells, most concern the tea leaves extract and its compounds- catechins with presence of the galloyl moiety as the most active, as well as of A-type proanthocyanidins from fruits of *Vaccinium* species. Other sources of proanthocyanidins such as grape seeds and traditional medicinal plants, were seldom. The in vitro studies proved their immunomodulatory activity, among others by influencing on immune cell regulation, proinflammatory cytokines synthesis and gene expression as well as by radical scavenging and inhibition of certain enzymes. They modulate NF-κB (nuclear factor kappa-light-chain-enhancer of activated B cells) and mitogen-activated protein kinase (MAPK) pathways. Despite these promising results there is still much less studies using animal models (11) and only a few clinical studies (3). In conclusion, the potential of flavan-3-ols and their derivatives in prevention and alleviation of periodontitis is remarkable but clinical evidence is urgently needed for issuing credible dietary recommendation and complementary treatments.

## Figures and Tables

**Table 1:** Immunomodulatory effects of proanthocyanidins (PAs) or flavan-3-ols on host cells and tissues—in vitro studies.

Active Compound/Extract/Fraction	Cells/Tissues	Methods	Results	Authors, Year	Ref.
Cranberry *(Vaccinium macrocarpon* Ait.) concentrate from capsules (Uriach-Aquilea OTC, Barcelona, Spain) containing 130 mg A-Type proanthocyanidins	Human gingival fibroblasts (HGF), human osteosarcoma-derived osteoblasts (SAOS-2 cell line), phorbol myristate-acetate (PMA)-induced macrophages (from THP-1 cells, a monocytic leukemia cell line).	All the tested cells were exposed for 24 h to different cranberry concentrates (25, 50, and 100 μg/mL). After 0, 3 and 7 days cell viability assay was performed. Interleukins: IL-8, IL-1β, IL-6, and IL-10 expression of lipopolysaccharide (LPS from *Escherichia coli*)-stimulated macrophages, and macrophage polarization were evaluated through determination of live-cell protease activity, enzyme-linked immunosorbent assay, and immunofluorescence staining semi-quantification.	After 24 h exposure, the HGF, SAOS-2, and macrophages viability was not reduced by the cranberry concentrates; Expression of proinflammatory IL-8 and IL-6 was downregulated by 50 µg/mL and 100 µg/mL PAs, but expression of anti-inflammatory IL-10 was upregulated at 100 µg/mL. No influence on expression of IL-1β was seen. Exposed LPS-stimulated macrophages to PAs significantly decreased M1 polarization and increased M2 polarization.	Galarraga-Vinueza et al., 2020	[12]
Unverified commercial proanthocyanidin (PA) purchased at ChemFace (Wuhan, China), with molecular weight = 594.52 and untypical structure	Human periodontal ligament fibroblasts (HPDLFs).	HPDLFs used in the study were treated by the tumor necrosis factor-alpha (TNF-α), PA, or their combination. The mineralization markers and osteogenic differentiation markers and associated markers were detected by quantitative real-time polymerase chain reaction (qRT-PCR), alizarin red S staining, and alkaline phosphatase (ALP) activity assay.	PA (0.1, 1, 10 μg/mL) significantly upregulated expression of osteogenesis-related genes and proteins and ALP activity in HPDLF compared with the control group in the non-inflammatory environment. PA (1 μg/mL) has significantly reversed the inhibition of osteogenesis-related gene and protein expression, ALP activity, and mineralization caused by TNF-α. PA could regulate osteogenesis of HPDLFs by suppressing the NF-κB signaling pathway.	Huang et al., 2020	[13]
Catechin	THP-1 cells.	THP-1 cells were pre-treated with catechin (40 µM) and then infected with bacteria *Porphyromonas gingivalis*. The cytokine levels (IL-1*β* and TNF-α) and relevant protein expression in THP-1 cells (e.g., pro-IL-1β, NF-κB, toll-like receptor: TLR2, TLR4, and mitogen-activated protein kinases (MAPK) were measured using an ELISA kits and Western blot analysis, respectively. Confocal laser scanning microscopy was used in this study to measure an apoptosis-associated speck-like protein containing a caspase recruitment domain (ASC) pyroptosome formation.	Catechin has inhibited *P. gingivalis*-induced the secretion of the TNF-α and IL-1*β* in tested THP-1 macrophages. Decreased production of IL-1*β* caused by catechin was due to its inhibition of pro-IL-1*β* expression via the downregulation of NF-κB, p38 MAPK, and TLR signaling. Moreover, the tested compound has inhibited the activation of inflammasomes induced by *P. gingivalis* but has not affected the growth of this bacteria.	Lee et al., 2020	[14]
*Pelargonium sidoides* DC root extract (PSRE) and proanthocyanidin (prodelphinidins) fraction from PSRE (PACN)	Rat gingival fibroblast cell culture; Bone marrow-derived macrophages (BMDM); Human peripheral blood mononuclear cells (PBMCs).	Cells (rat gingival fibroblast, BMDM or PBMCs) were treated with PACN, PSRE, and LPS and in case of BMDM, with IFNγ. After treatments medium was collected and assayed for TNF-α, IL-6, IL-8 and prostaglandin E_2_ (PGE2) production using TNF-α mouse, IL-6 human IL-8 rat and PGE2 rat; The ability of PSRE, PACN to modulate IL-1*β*, TNF-α and iNOS expression in BMDM and PBMCs was evaluated. Expression of proinflammatory cell surface markers CD80 and CD86 was analyzed by the flow cytometry after 24 h treatment LPS + interferone-γ (IFNγ)-activated BMDM with PACN or PSRE.	PSRE and PACN (50–100 µg/mL) has been found to suppress the LPS-induced IL-8 and PGE_2_ release from the tested gingival fibroblasts and IL-6 release from mononuclear leukocytes. PACN has demonstrated a slightly stronger IL-8 and IL-6 release suppressing activity, and significantly stronger PGE2 release suppressing activity than PSRE. PSRE and PACN (100 µg/mL) has significantly suppressed the mRNA transcription of IL-1β, iNOS, and COX-2, but not TNF-α. PSRE and PACN (100 µg/mL) reduced the level of CD80 and CD86-positive cells. PSRE reduced by 58% and PACN by 71% the population of cells with the exposed markers, compare to LPS + IFNγ-activated BMDM without the treatment.	Jekabsone et al., 2019	[15]
Commercial green and black tea extracts, epigallocatechin-3-gallate (EGCG), theaflavin fraction (mixture of theaflavin, theaflavin-3-gallate, theaflavin-3′-gallate, and theaflavin-3, 3′-digallate) (80% purity)	U937 human monocytes differentiated to macrophage-like cells, gingival keratinocyte cell line B11, human oral epithelial cell line GMSM-K.	Black and green tea extract, EGCG, or theaflavins were used to treat the macrophage-like cells prior to being stimulated with 10 ng/mL or 100 ng/mL of recombinant human TNF-α. Intracellular and released IL-1β levels were quantified using an ELISA kit. Caspase-1 activation and quantification as well activation of the NF-κB signaling pathway were determined by commercial assay. Gingival keratinocytes were used to study the influence of tea-polyphenols on the TNF-α-induced disruption of tight junction integrity which was determined by measuring TER (transepithelial electrical resistance). ELISA kit was used to quantify IL-8 secretion by oral epithelial cells.	The extracts from a green tea and black tea, EGCG and theaflavins in range 7.9–62.5 μg/mL were found to significantly and dose-dependently reduced secretion IL-1β by TNF-α -treated macrophages (at 62.5 μg/mL all substances inhibited the secretion of IL-1β by more than 94%, except black tea—64.5%), as well as reduced the activation of caspase-1 and NF-κB activation. Green tea extract, theaflavins, EGCG and to a lesser extent, black tea extract protected keratinocytes against the TNF-α-mediated breakdown of barrier integrity. The treatment of keratinocytes with tea polyphenols markedly mitigated the morphological changes of tight junction proteins such as occludin and zonula occludens-1. At a concentration of 62.5 μg/mL, the green tea extract, EGCG, and theaflavins reduced the secretion of IL-8 by 93.1%, 98.8%, and 70.8%, respectively. A much higher concentration of black tea extract (250 µg/mL) was required to reduce the secretion of IL-8 (78%).	Ben Lagha and Grenier, 2019	[16]
Cranberry proanthocyanidins (PAs) isolated from cranberries (*Vaccinium macrocarpon*)	U937 human monocytes differentiated to macrophage-like cells.	Adherent macrophages were exposed to leukotoxin LtxA (1 µg/mL) depending of the presence of cranberry PAs. A RealTime-Glo™MT Cell Viability Assay was performed. The ELISA kit was used to quantify the amounts of IL-1β, IL-18, and caspase-1 secreted into the culture medium or contained in the macrophages. The P2X7 receptor and cryopyrin (CIAS) mRNA expression was determined by q RT-PCR analysis. A total reactive oxygen species (ROS)/superoxide detection kit was used to measure intracellular ROS production. Influence of cranberry PAs on binding of FITC–LtxA to macrophages was examined by flow cytometry.	Cranberry PAs substantially reduced the cytotoxic effects of LtxA on macrophages. At the 125 µg/mL concentration of the cranberry Pas, the release of caspase-1, IL-1β, and IL-18 was significantly reduced (by 100%, 99.3%, and 98.7%, respectively), compared to cells treated with LtxA alone. However, this strong reduction was already seen with lower concentration—15.625 µg/mL of PAs (about 80–90% reduction—as can be read from the graph). In contrast, the intracellular levels of these cytokines were comparable to those of control cells. 125 µg/mL of cranberry PAs reduced the expression of P2X7 and CIAS by 45.8% and 30.2%, respectively, as well reduced the ROS and superoxide production by 92.2% and 72.7%, respectively, compared to the control (macrophages exposed only to LtxA). 62.5 µg/mL and125 µg/m of cranberry PAs blocked the binding of FITC-LtxA to macrophages by about 50%.	Ben Lagha et al, 2019	[17]
The buds of *Castanopsis lamontii* Hance water extract (CLE) rich in epicatechin and procyanidin B2; epicatechin (EC); procyanidin B2 (PB2)	Mouse macrophage RAW264.7 cells.	The RAW264.7 cells were cotreated or pretreated with LPS and CLE/EC/PB2 and then the expression of TLR-4 pathway-related proteins (TLR-4, p-NF-κB (p65), iNOS, and COX-2) and the release of NO, PGE2, and TNF-α were determined. The concentration of NO, PGE2, TNF-α was measured using appropriate test kits The Western Blot (WB) assay was used in the study to determine the expression of TLR-4, p-NF-κB (p65), iNOS, and COX-2.	CLE (400 μg/mL) and two compounds-PB2 (34.4 µg/mL) and EC(120 µg/mL) (equivalent to the concentration of PB2 and EC in 400 µg/mL CLE) significantly decreased the release of NO, PGE2, and TNF-α from LPS-stimulated macrophages in LPS cotreated and pretreated group as well as decreased Lipopolysaccharide-stimulated up-regulation of TLR4, p-NF-κB (p65), COX-2, and iNOS in RAW264.7 cells. Compared with EC, PB2 was much more potent in suppressing the LPS-stimulated inflammatory response.	Gao et al., 2019	[18]
Highbush blueberry (*Vaccinium corymbosum* L.) proanthocyanidins (PAs)	U937 human monocytes differentiated to macrophage-like cells.	In the study adherent macrophage like cells were pre-treated with the PAs and then stimulated with *A. actinomycetemcomitans* LPS. ELISA was used to quantify the secretion of pro-inflammatory cytokines (IL-1β, TNF-α, IL-6 and C-X-C motif chemokine ligand CXCL8) and MMPs (MMP-3 and MMP-9). NF-κB activation was monitored by using the U937-3xκB-LUC monocyte cell line transfected with a luciferase reporter gene. MTT assay was used to determine viability of cells treatment with PAs.	PAs significantly and dose-dependently decreased secretion of pro-inflammatory cytokines from LPS-stimulated macrophages. PAs at 125 µg/mL reduced the secretion of IL-1β, TNF-α, IL-6, and CXCL8 by 75.34%, 81.64%, 48.27%, and 90.19%, respectively, whereas MMP-9 and MMP-3 secretion was attenuated by 68.78% and 93.04%, respectively. The PAs were also found to inhibit the activation of the nuclear factor-κB (NF-κB) signaling pathway.	Ben Lagha et al., 2018	[19]
Mixture of theaflavins (TFs) from black tea (theaflavin-3-gallate, theaflavin-3′-gallate and theaflavin-3-3′-digallate, with more than 80% purity)	Gingival keratinocyte cell line, B11; U937 human monocytes differentiated to macrophage-like cells.	Adherent macrophage like cells were pretreated with the TFs and stimulated with *P. gingivalis*. Secreted matrix metalloproteinase (MMP-3, MMP-8, MMP-9) and pro-inflammatory cytokines (IL-1β, TNF-α, IL- 6, and CXCL8) were then quantified by ELISA. Effect of theaflavins (TFs) on the activity of MMP-9 was monitored using a fluorogenic assay. NF-κB activation was monitored by using the U937-3xκB-LUC monocyte cell line transfected with a luciferase reporter gene. MTT assay was used to determine viability of cells treatment with TFs.	At a concentration of 125 µg/mL, the TFs have reduced the secretion of Interleukin 1 beta by 98.4%, TNF-α by 98.8%, Interleukin 6 by 97.7% and of CXCL8 by 84% compared to the controls as well reduced the secretion of MMP-3 by 97.3%, MMP-8 by 99.9%, MMP-9 by 95.7%. The TFs mixture at 125 µg/mL reduced MMP-9 activity by 100%. The TFs inhibited the activation of the NF-κB signaling pathway.	Ben Lagha and Grenier, 2017	[20]
80% methanol extract of green tea (*Camellia sinensis* (L.) Kuntze) and commercially purchased epigallocatechin-gallate (EGCG)	Human neutrophils.	Methanol extract and EGCG were tested in vitro environment for their ability to inhibit MMP-9 activity and/or its release from neutrophils using a b-casein cleavage assay and gelatin zymography, respectively.	Methanol extract and EGCG at 0.1% (*w/v*) has completely inhibited the activity of matrix metalloproteinase-9, as well significantly inhibited the release of MMP-9 from formyl-Met-Leu-Phe-OH (FMLP) stimulated human neutrophils by 62.01% and 79.63%, respectively and from unstimulated neutrophils (52.42% and 62.33%, respectively).	Kim-Park et al., 2016	[21]
70% ethanolic blueberry extract (*Vaccinium angustifolium* Ait.*)*—phenolic acids, flavonoids and procyanidins made up 16.6%, 12.9%, and 2.7% of the blueberry extract, respectively	U937 human monocytes differentiated to macrophage-like cells.	The macrophage like cells were pretreated with the blueberry extract and then stimulated with *F. nucleatum.* ELISA kits were used to quantify IL-1β, IL-6, CXCL8, TNF-α, MMP-8, and MMP-9 concentrations. Activity of MMP-9 was monitored using fluorogenic assay. The ability of the blueberry extract to inhibit the NF-κB signaling pathway in U937-3xκB cells was evaluated in the study.	The blueberry extract was found to dose-dependently inhibit the activation of NF-κB induced by *Fusobacterium nucleatum*. A pre-treatment of macrophages with the tested blueberry extract (62.5 μg/mL) has inhibited the secretion of interleukin 1 beta, TNF-α, and interleukin 6 by 87.3%, 80.7%, and 28.2%, respectively. The secretion of the chemokine CXCL8 was not affected by 62.5 μg/mL of the tested blueberry extract, but 500 μg/mL, 250 μg/mL, or 125 μg/mL extract have decreased the secretion of the CXCL8 by 79%, 57.9%, and 11.2% respectively. The secretion of MMP-8 and MMP-9 was also dose-dependently inhibited as well MMP-9 activity.	Ben Lagha et al., 2015	[22]
type-A cranberry proanthocyanidins (AC-PAs) and epigallocatechin-3-gallate (EGCG)	A 3D co-culture model composed of gingival fibroblasts embedded in a collagen matrix and overlaid with gingival epithelial cells.	The 3D co-culture model treated with the concentrations of AC-PAs that are not cytotoxic (25 or 50 μg/mL), EGCG (1 or 5 μg/mL) and LL-37 (peptide cathelicidin) individually and in combination (AC-PAs+LL-37 and EGCG+LL-37) were stimulated with LPS from the *A. actinomycetemcomitans*. Multiplex ELISA assays were used to quantify the secretion of 54 host factors, among them were chemokines, cytokines, growth factors MMPs, and tissue inhibitors of metallopeptidases (TIMPs).	From the forty one different cytokines, chemokines, and growth factors that were analyzed in this study, LPS from the *A. actinomycetemcomitans* has significantly increased the secretion only granulocyte colony-stimulating factor (G-CFS), GRO-a (CXCL 1), IL-6, IL-8, IP-10, and monocyte chemoattractant protein (MCP-1) by the 3D co-culture model compared to the unstimulated control. When used individually, 25 μg/mL of AC-PAs significantly reduced the secretion of G-CFS (42%), GRO-a (33%), interleukin-8 (39%), IP-10 (72%), and MCP-1 (72%), but had no significant effect on the secretion of interleukin-6, while EGCG at 5 μg/mL significantly reduced the secretion of GRO-a (13%), IL-8 (34%), IP-10 (22%), and MCP-1 (70%), but had no significant effect on the secretion of G-CSF and interleukin-6. AC-PAs and LL-37 acted in synergy to reduce GRO-a, G-CSF, and IL-6 and had an additive effect on reducing the secretion of IL-8, IP-10, MCP-1. EGCG and LL-37 acted in synergy to reduce the secretion of GRO-a, G-CSF, interleukin-6, interleukin-8, and IP-10, and had an additive effect on MCP-1 secretion. None of the concentrations of AC-PAs and EGCG tested, individually or in combination, decreased LPS-stimulated secretion of MMPs (-1, -2, -3, -7, -8, -9, -10, -12 and -13) or TIMPs (-1, -2, -3 and -4) from 3D co-culture model.	Lombardo Bedran et al. 2015	[23]
Commercial black tea extract (with theaflavin content of 40.23%); theaflavin, theaflavin-3,3′-digallate, epigallocatechin-3-gallate EGCG	The oral epithelial cells (OBA-9).	The epithelial cells were pretreated with the extract form black tea, theaflavin, theaflavin-3,3′-digallate or EGCG prior to being stimulated with *A. actinomycetemcomitans* lipopolysaccharide. ELISA assays were used to quantify the secretion of IL-8, human β-defensins (hBD-1, hBD-2 and hBD-4) by OBA-9 cells.	The extract obtained from the black tea (200 μg/mL), as well as theaflavin (50 μg/mL) and theaflavin-3,3′-digallate (50 μg/mL) reduced IL-8 secretion by 85%, 79%, and 86%, respectively. EGCG used as a positive control reduced IL-8 little stronger. The secretion of all 3 hBD antimicrobial peptides was up-regulated dose-dependently. Only theaflavin did not induce the secretion of significant amounts of hBDs from OBA-9 cells.	Lombardo Bedran et al., 2015	[24]
The commercial green tea extract with polyphenol content ≥98%, including 45% epigallocatechin-3-gallate EGCG	The immortalized human gingival epithelial cell line, B11.	Immortalized human gingival cells from epithelium were treated with various amounts of extract from green tea or EGCG (from 25 μg/mL up to 200 μg/mL). ELISA was used to measure the secretion of hBD1 and hBD2 and real-time PCR was used to evaluate their gene expression. The ability of tested tea extract and EGCG to prevent hBD degradation by *Porphyromonas gingivalis* was evaluated by ELISA.	Tested extract from green tea and EGCG dose-dependently induced the secretion of hBD1 and hBD2 from gingival epithelial cells. They increased expression of the hBD gene in tested epithelial cells. Green tea or EGCG-induced secretion of hBD1 and hBD2 appeared to involve ERK1/2 and p38 MAPK. Green tea extract and EGCG prevented the degradation of recombinant hBD1 and hBD2 by a culture supernatant of *P. gingivalis.*	Lombardo Bedran et al., 2014	[25]
Non-dialyzable material (NDM) prepared from concentrated cranberry (*Vaccinium macrocarpon*) juice, containing 65.1% proanthocyanidins.	The Smulow-Glickman (S-G) human gingival epithelial cell line.	S-G cells were incubated with IL-1β in the presence or absence of NDM or inhibitors of NF-κB-NBD or AP-1-SP600125. The IL- 6 levels were measured by ELISA kit. Effects of NDM on IL-1β-activated NF-κB and AP-1 and phosphorylated intermediates in both pathways were measured by ELISAs.	Production of IL-6 was increased in IL-1β stimulated S-G cells. NDM was stronger inhibitor of IL-6 production in IL-1β stimulated S-G cells than either NBD peptide or SP600125 alone and was similar to the combination of these inhibitors. IL-1β stimulated NF-κB and AP-1 activation was inhibited by NDM. However, NDM did not affect IL-1β-stimulated levels of phosphorylated intermediates in the NF-κB pathway (IκBα) or the AP-1 pathway (c-Jun, ERK1/2).	Tipton et al., 2014	[26]
Same as above	The Smulow-Glickman (S-G) human gingival epithelial cell line; normal human gingival fibroblasts.	S-G and normal human gingival fibroblasts were incubated with NDM, IL-17, or NDM+IL-17. IL-6 and IL-8 in culture supernatants were measured by ELISA.	In both cell lines, IL-17 has been found to significantly stimulate the production of IL-6 and IL-8. Non cytotoxic levels of NDM (5–50 μg/mL) inhibited constitutive IL-6 and IL-8 production as well their IL-17-stimulated cytokine production by epithelial cells and fibroblasts.	Tipton et al., 2013	[27]
Same as above	Human gingival fibroblast cell line derived from patient with aggressive periodontitis (AgP); Normal human gingival fibroblast cell lines (GN23, GN56, GN60).	AgP or normal fibroblasts were incubated with NDM or LPS (from *Fusobacterium nucleatum* or *Porphyromonas gingivalis*) ± NDM. The cell viability and membrane damage were tested by MTT assay and enzyme activity released into cell supernatant, respectively. ELISA was used to measure IL-6 and MMP-3 secretion. Nuclear p65 levels were measured using a colorimetric assay.	NDM ≤ 100μg/mL showed no significant effect on tested AgP fibroblast viability, but higher concentration decreased their viability. No membrane damage was seen after short-term exposure to NDM, or LPS ± NDM. NDM (50 μg/mL) inhibited LPS-stimulated nuclear p65 levels (by 25% for LPS from *F. nucleatum* and by 80% for LPS from *P. gingivalis*) as well inhibited constitutive or LPS-stimulated MMP-3 in AgP fibroblasts. NDM increased IL-6 in LPS-stimulated AgP fibroblast but decreased in normal human gingival fibroblast.	Tipton et al., 2013	[28]
Non-dialyzable material (NDM) prepared from concentrated cranberry (*Vaccinium macrocarpon*) juice, rich in proanthocyanidins	RAW 264.7 mouse macrophages.	RAW 264.7 mouse macrophages were exposed to culture media *(P. gingivalis* and *F. nucleatum)* with or without NDM (4 mg/mL). The secreted form of mouse TNF-α was quantified using two-site ELISA. Macrophage functionality was investigated using a phagocytosis assay.	NDM completely inhibited bacteria’s increased expression of TNF-α in macrophages without reducing their viability. Furthermore, the addition of NDM caused increasing the phagocytosis of *P. gingivalis* (from 10% to 20%)*,* and little reduction in the phagocytosis of *F. nucleatum* (from 50 to 40%).	Polak et al., 2013	[29]
(-)-Epigallocatechin gallate (EGCG)	Human gingival fibroblasts (HGFs).	HGFs were cultured in the presence or absence of EGCG (3.125–50 µg/mL) prior to their incubation with IL-1β + IL-4 or TNF-α + IL-4-stimulations, then the CCL11 concentrations were measured with ELISA. Western blot analysis was used in the study to confirm the effects of EGCG on IL-1β + IL-4 or TNF-α + IL-4-induced phosphorylation of signal transduction molecules.	IL-4 was found to synergistically enhance CCL11 production in IL-1β or TNF-α-stimulated HGFs. EGCG reduced production of CCL11 in IL-1β/IL-4 or TNF-α/IL-4-stimulated HGFs, in a concentration dependent manner. CCL11 production in HGFs was positively regulated by p38 MAPK, ERK, and JNK. EGCG prevented activation of ERK and JNK, but not p38 MAPK, induced by IL-1β/IL-4 or TNF- α/IL-4-in HGF.	Hosokawa et al., 2013	[30]
A-type cranberry proanthocyanidins (APAC) and licochalcone A (LA)-chalcone, not proanthocyanidin	U937 human monocytes differentiated to macrophages.	IL-1β, TNF-α, IL-6, and IL-8 production by macrophages treated with the APAC (or/and LA) and stimulated by *A. actinomycetemcomitans* LPS was evaluated by ELISA kits. Influence of APAC (or/and LA) on MMP-9 and *Porphyromonas gingivalis* collagenase activities was measured using fluorometric assays. Macrophages viability was evaluated with MTT assay.	ACPAC in 25 µg/mL or 50 µg/mL concentration reduced the LPS-induced secretion of TNF-α, IL-6 and IL-8 in a macrophage model, but not IL-1β. A reduction in secretion of IL-1β was seen when ACPAC was used together with LA. ACPAC (25 µg/mL) inhibited MMP-9 activity by 32% and *P. gingivalis* collagenase by 66%.	Feldman and Grenier, 2012	[31]
AC-PAs fraction from cranberries *(Vaccinium macrocarpon)*	Human osteoclast precursor cells.	This study investigated the influence of the AC-PAs on osteoclast formation and bone resorption. The degree of osteoclast formation was assessed by quantification of TRAP-positive stained multinucleated cells, while the secretion of IL-8 and MMP-2, MMP-9 was measured with ELISA. Bone resorption was evaluated using a human bone plate coupled with an immunoassay that detected the release of collagen helical peptides. MTT assay was used to measure the cytotoxic effect of AC-PAs on osteoclastic cells.	AC-PAs at 10 μg/mL, 25 μg/mL, and 50 μg/mL caused a 38%, 84%, and 95% inhibition of RANKL-dependent osteoclast differentiation, respectively. AC-PAs increased the secretion of IL-8 and inhibited the secretion of both MMP-2 and MMP-9 in a dose-dependent manner. AC-PAs significantly decreasing the release of collagen helical peptides suggested that can prevent bone resorption. AC-PAs did not exhibit any toxic effect on osteoclastic cells from 10 μg/mL to 100 μg/mL.	Tanabe et al., 2011	[32]
50% EtOH extract from *Myrothamnus flabellifolia* Welw. (MF)	KB cells (ATCC CCL-17, HeLa).	KB cells were pretreated with MF (10 µg/mL and 100 µg/mL) and infected with *P. gingivalis*. The cytokine gene expression was monitored using RT-qPCR and IL-6 level using ELISA.	10 and 100 µg/mL of MF significantly decreased (upregulated by *P. gingivalis)* gene expression for IL-1β, IL-8 and TNF-α, but not IL-6 compare to control cells (not exposed to tested extract). However, preincubation of the KB cells with MF before exposure to *P. gingivalis* resulted in significant lower concentration of IL-6 in the cells than in MF-untreated control group.	Löhr et al., 2011	[33]
Epigallocatechin-3-gallate (EGCG) and epicatechin-3-gallate (ECG)	Human dental pulp cells (HDPC).	HDPC were pretreated with or without EGCG or ECG (1–50 µg/mL) for 1 h, and incubated with *E. coli* LPS (1 µg/mL) or *S. aureus* PG (10 µg/mL) for 4 h or 24 h. After incubation, the quantities of IL-6 and IL-8 (by ELISA) were determined, and the attached cells were used for RNA extraction (gene expression of IL-6 and IL-8 using the RT-qPCR) or flow cytometric analysis (for expression of intercellular adhesion molecule-1 (ICAM-1) and of vascular cell adhesion molecule-1 (VCAM-1)).	Treatment with EGCG and ECG significantly reduced, in a concentration-dependent manner, IL-6 and IL-8 mRNAs and the respective proteins level in dental pulp cells exposed to LPS or PG. Up-regulated ICAM-1 or VCAM-1 expression on LPS or PG stimulated HDPC was decreased by treatment with EGCG or ECG.	Nakanishi et al., 2010	[34]
Epigallocatechin gallate (EGCG) and epicatechin gallate (ECG)	Human gingival fibroblasts (HGFs) isolated from healthy gingiva.	HGFs cultured in the presence or absence of EGCG or ECG prior to their incubation with Oncostatin M (OSM), then the CXCL10 concentrations of the culture supernatants were measured with ELISA. The effects of EGCG and ECG on the p38 MAPK, JNK, Akt, and STAT3 phosphorylation induced by OSM in HGFs was measured using Western blotting analysis with antibodies. The effects of EGCG or ECG on OSMRβ expression on HGFs were measured using flow cytometry.	EGCG or ECG (50 μg/mL) significantly inhibited (about 60%) the CXCL10 production induced by OSM treatment. EGCG (50 μg/mL) significantly prevented OSM induced phosphorylation of JNK, Akt (Ser473) and STAT3 (Tyr705 and Ser727), whereas ECG (50 μg/mL) prevented phosphorylation of JNK and Akt (Ser473). EGCG and ECG attenuated OSMRβ expression on HGFs.	Hosokawa et al., 2010a	[35]
Epigallocatechin gallate (EGCG), epicatechin gallate (ECG), theaflavin-3,3′-digallate (TFDG)	Human gingival fibroblasts (HGFs) isolated from healthy gingiva.	HGFs were cultured in the presence or absence of EGCG, ECG, and TFDG (5 μg/mL or 50 μg/mL) prior to their incubation with TNF superfamily member 14 (TNFSF14), then the IL-6 level in the culture supernatants were measured by ELISA. The effects of TFDG, ECG, EGCG, on MAPKs and NF-κB pathways in TNFSF14-stimulated HGFs were measured using Western blotting analysis. The effect of TFDG, ECG, EGCG, on TNFSF14 receptor expression (HVEM and LTbR) in HGFs were measured using flow cytometry.	Using 50 μg/mL of TFDG, ECG, or EGCG has significantly lowered the IL-6 production in TNFSF14-stimulated HGFs, without harming cells. TFDG, ECG or EGCG inhibited TNFSF14-induced ERK, JNK, and NF-κB activation and suppressed TNFSF14 receptor expression in HGFs. It is supposed that TFDG, ECG or EGCG suppressed IL-6 production in TNFSF14-stimulated HGFs through the inhibition of JNK, ERK, or NF-κB activation.	Hosokawa et al., 2010b	[36]
Type-A Cranberry Proanthocyanidins (AC-PAs) were isolated from cranberries (*Vaccinium macrocarpon*)	Oral epithelial cells (GMSM-K).	Increasing concentrations of AC-PAs (25 µg/mL to 100 µg/mL) were used to pretreat epithelial cells before their stimulation with *P. gingivalis*. ELISA kits were used to quantify IL-6 and IL-8, CCL5 concentrations in the free-cell supernatants. AC-PAs influence on NF-κB p65 activation was investigated.	Type-A Cranberry Proanthocyanidins have significantly decreased the secretion of IL-8 and CCL5 at all of the concentrations tested in a dose-dependent manner, where 100 µg/mL dose has reduced secretion of IL-8 and CCL5 by more than 80% and was not related to loss of cell viability. AC-PAs did not affect the secretion of IL-6. 50 µg/mL of AC-PAs significantly decreased the *P. gingivalis*-induced activity of NF-κB p65 from 203.9% to 91%.	La et al., 2010	[37]
Same as above	Human monocyte-derived macrophages.	Investigate the effects of Type-A cranberry PAs (25 µg/mL, 50 µg/mL, and 100 µg/mL) on: (1) The production of various MMPs by human monocyte-derived macrophages stimulated with LPS from *A. actinomycetemcomitans* by using ELISA combined with piezoelectric printing technology, (2) the catalytic activity of recombinant MMP-9 and MMP-1, and (3) the expression of 5 protein kinases and the activity of NF-κB p65 in LPS stimulated macrophages using commercial kits. Cytotoxicity was determined using MTT assay.	No toxic effects toward macrophages were detected following a 24 h treatment with a 100 µg/mL of tested proanthocyanidins. AC-PAs significantly reduced the production of MMP-7, MMP-8, and MMP-13 in LPS stimulated macrophages at all tested concentrations, whereas production of MMP-3 was reduced significantly only at the highest concentration (100 µg/mL) and production of MMP-1, MMP-9 at 50 µg/mL. AC-PAs also significantly inhibited the catalytic activity of MMP-1 and MMP-9. Inhibition of MMP production was associated with inhibition of the NF-κB p65 activity and decreased phosphorylation of key intracellular kinases.	La et al.,2009	[38]
Epigallocatechin-3-gallate (EGCG)	MG-63, a human osteosarcoma cell line.	MG-63 cells were incubated with OSM (oncostatin M) alone or together with 10 µg/mL EGCG (prior to addition of OSM). The levels of Cyr61 were measured using Western blot analysis. Moreover, MG-63 cells were treatment with Cyr61 and level of CCL2 was measured with an ELISA kit.	OSM was found to stimulate Cyr61 synthesis in MG-63 cells in a time dependent manner, whereas EGCG has significantly attenuated this effect. Treatment of MG-63 cells with Cyr61 resulted in increased release of CCL2.	Lee et al., 2009	[39]
Apple condensed tannin (ACT) isolated from apple. Hop bract polyphenols (HBP) fraction rich in proanthocyandins. HMW-HBP (high molecular weight fraction) and LMW-HBP (low molecular weight fraction) separated from HBP. Epigallocatechin-3-gallate (EGCG)	Human gingival epithelial (HGE) cells.	HGE cells were stimulated with *P. gingivalis* membrane vesicles (50 µg/mL of final concentration) in the presence or absence of ACT, HBP, HMW-HBP, LMW-HBP, EGCG, or 3 isolated compounds from LMW-HBP: 2-(2-methylpropanoyl)-phloroglucinol 1-O-b-D-glucopyranoside (MPPG), isoquercitrin, and astragalin at various concentrations (5–25 µg/mL) for 24 h. PGE_2_ secreted in the culture supernatant was quantified using an ELISA kit.	The strongest, significantly inhibition of PGE_2_ production was seen for EGCG, starting from 10 µg/mL. ACT did not influence on the PGE2 production. HBP and LMW-HBP significantly and moderately inhibited the production of PGE_2_, similar like isolated from LMW-HBP compounds: MPPG, isoquercitrin, astragalin.	Inaba et al., 2008	[40]
Fraction from cranberries (*Vaccinium macrocarpon*), obtained after dialysis; Non-dialysable material (NDM) contains 65.1% proanthocyanidins	Human gingival fibroblasts (HGF-1), U937 human monocytes differentiated to macrophages.	HGF-1 and macrophages were treated with the NDM and then stimulated with LPS from *A. actinomycetemcomitans.* MMP-9 and MMP-3 production was measured using ELISA kits. Elastase, MMP- 9 and MMP-3 activities in the presence of the NDM were tested by using colorimetric or fluorogenic substrates. The antibody microarrays were used to characterize changes in the expression and phosphorylation state of fibroblast signaling proteins. MTT assay was used for evaluated cells viability.	Production of MMP-9 and MMP-3 by LPS stimulated macrophages pretreated with the NDM were inhibited significantly, in a dose dependent manner, similarly production of MMP-3 by fibroblast. However, MMP-9 response in LPS stimulated fibroblast wasn’t observed. Cranberry fraction wasn’t toxic towards fibroblast and macrophages. NDM revealed inhibitory effect on some fibroblast intracellular signaling proteins (Fos, JNK, Jun, MKK3/6, MKK6, Rac1/cdc42, and ROCK2). Elastase, MMP-9 and MMP-3 activities were significantly inhibited by NDM even at low concentration—10 µg/mL (about 50%).	Bodet et al., 2007	[41]
Same as above	Human gingival fibroblasts HGF-1.	IL-6, IL-8, and PGE2 production by fibroblasts treated with the NDM (10, 25 or 50 µg/mL) and stimulated by LPS from *A. actinomycetemcomitans* was evaluated by ELISA. Changes in the expression and phosphorylation state of fibroblast intracellular signaling proteins were characterized by antibody microarrays. Fibroblast viability was evaluated using the MTT assay.	The production of PGE2, IL-8 and IL-6 by LPS stimulated fibroblasts was inhibited by NDM at non-toxic concentrations of 10–50 µg/mL. At a final concentration of 50 µg/mL cranberry fraction completely inhibited the IL-8 production, whereas a 72% inhibition was noted at a concentration of 10 µg/mL. The PGE2 and IL-6 production was significantly reduced in range 25 and 50 µg/mL. The results suggest that the cranberry fraction can act by reducing the AP-1 activity. NDM also reduced expression of COX-2.	Bodet et al., 2007	[42]
Water–alcohol grape seed extract (GSE) from red grape seeds containing 95% oligomeric proanthocyanidins (PAs), gallic acid (GA), epigallocatechin gallate (EGCG)	The murine macrophages cell line RAW 264.7.	Cells were preincubated with non-cytotoxic concentrations of GA (4 μg/mL), EGCG (0.5 μg/mL) or GSE (4 μg/mL) and stimulated with LPS of *A. actinomycetemcomitans*, *F. nucleatum*. NO production was quantified using the colorimetric Griess assay, iNOS expression was evaluated using immunoblotting, whereas ROS production was measured with the fluorescent 123-dihydrorhodamine dye.	GSE as well EGCG strongly decreased ROS and NO production as well iNOS expression in LPS-stimulated macrophages. GA also showed a strong inhibitory effect on NO production; however, without affecting iNOS expression and slightly increasing ROS production.	Houde et al., 2006	[43]
EGCG (−)-epigallocatechin gallate	Mouse calvarial primary osteoblastic cells.	Mouse calvarial primary osteoblastic cells were pretreated with epigallocatechin gallate (20 µM) in the presence of sonicated *Porphyromonas gingivalis* extracts. The effect of epigallocatechin gallate on the gene expression of MMPs was examined by RT-PCR. The effect of EGCG on osteoclast formation was confirmed with TRAP staining in a co-culture system of mouse calvarial primary osteoblastic cells and bone marrow cells.	*P. gingivalis* stimulated only the expression of MMP-9 mRNA (215% increase) and this effect was significantly reduced by tested EGCG, reaching the level of MMP-9 mRNA expression as in untreated cells. Neither *P. gingivalis* extracts nor EGCG influenced the transcription levels of MMP-2 and MMP-13. Osteoclast formation was significantly inhibited by EGCG in the co-culture system.	Yun et al., 2004	[44]
Fraction of green tea polyphenols (GTP), (−)epigallocatechin gallate (EGCG), (−)-epicatechin gallate (ECG), (−)-epigallocatechin (EGC), (−)-epicatechin (EC), (+)-catechin (C)	Partially purified MMP-12 from the conditioned medium of the mouse macrophage cell line NCTC 3749, human proMMP-2 and mouse proMMP-9 were activated with APMA (aminophenyl-mercurin acetate).	GTP and 5 catechins were tested for their ability to inhibit MMP-12, MMP-9 and (MMP)-2 activities what was measured using fluorimetry and with gelatin or casein zymography. In addition, the activation of proMMP-2 by the lectin concanavalin A was determined after GTP exposure.	IC_50_ values for the inhibition of MMP-2 and MMP-9 activities were 10 µg/mL and 0.6 µg/mL for GTP, 95 µM and 28 µM for ECG and 6 µM and 0.3 µM for EGCG, respectively. MMP-12 was inhibited by more than 60% by 1 µM of ECG or EGCG. MMP-2, MMP-9 and MMP-12 activities were unaffected by C, EC, and EGC. MMP-2 activation by concanavalin A was reduced by 50% at 17.5 µg/mL of GTP and was almost completely inhibited at 35 µg/mL. Among catechins (at 100 µM), only EGCG inhibited the activation of MMP-2, The activation of proMMP-2 was inhibited in a dose-dependent manner. The complete abolition of the activation of pro-MMP-2 (induced by Con A) was recorded at 25 uM EGCG.	Demeule et al., 2000	[45]
Elm extract (EE) (n-butanol fraction from extract of *Ulmi macrocarpi cortex*) containing 20% of procyanidins) and the mixture of procyanidin oligomers (PO)	Gingival crevicular fluid (GCF) collected from periodontitis patients; Cultures of periodontal ligament (PDL) cells treated with *Treponema lecithinolyticum.*	The inhibitory effect of EE and PO on the MMPs in GCF (mostly MMP-8 and MMP-9) were assessed using gelatin zymography. The MMP-2 was verified by immunoblotting. Effects of EE and PO on cell proliferation were tested with MTT assay.	EE and PO inhibited activity of MMPs in GCF (the most abundant in MMP-8 and MMP-9), as well pro and active forms of MMP-2. PO was more effective than the EE. The IC_50_ values of the EE were 29 and 45 µg/mL for GCF collagenases (mostly MMP-8 and MMP-9 detected in GCF) and MMP-2, respectively. The corresponding IC_50_ values of the PO were 25 and 33 µg/mL, respectively. Contrary to PO, the EE extract at concentrations of 0.05–0.1% exhibited a cytotoxic effect towards PDL cells.	Song et al., 2003	[10]
Ethyl acetate fraction from the tea leaf (*Camellia sinensis.*) (+)-catechin (C), (−)-epicatechin (EC), (+)-gallocatechin (GC), (−)-epigallocatechin (EGC), (−)-epicatechin gallate (ECg), (−)-epigallocatechin gallate (EGCG).	Gingival crevicular fluid (GCF) collected from periodontitis patients; purified collagenase; in vitro study.	Ethyl acetate fraction and 6 isolated catechins were tested for their ability to inhibit purified collagenase activities using collagenase of *Clostridium histolyticum* and supernatant of *Porphyromonas gingivalis* as well collagenase activity in GCF. Collagenase activity was determined using a commercially available kit.	The complete inhibition of the collagenase activity was achieved by using a 100 µg/mL of ethyl acetate fraction from tea and 50 µg/mL ECG, EGCG. Other catechins, without the gallate residue had no effect on collagenase.	Makimura et al., 1993	[46]

**Table 2:** In vivo studies reporting influence proanthocyanidins or flavan-3-ols on periodontitis in animal model.

Active Compound/Extract/Fraction	Animal Model	Methods	Results	Authors, Year	Ref.
Catechin	Six-week-old C57BL laboratory mice were divided into 4 equal groups: (a) untreated normal control group; (b) *Porphyromonas gingivalis* infected group (c) *Porphyromonas gingivalis* infected + catechin group, (d) only catechin treated group.	Catechin (40 mg/kg body weight) was administrated orally to the animals 30 min before the *Porphyromonas gingivalis* injection, for two weeks, and subsequently every two days for an additional two more weeks. The animals were sacrificed after 49 days from the beginning of the study. Quantitative analysis of alveolar bone loss was performed using a microcomputed tomography.	The bone loss area was reduced significantly in the *Porphyromonas gingivalis*+catechin group (c), compared to the periodontitis group (b).	Lee et al., 2020	[14]
Water extract of Japanese green tea	8-week male C57BL/6 mice. 10 groups (*n* = 6 each) including control group with distilled water and 9 experimental groups with distilled water or different concentrations of Japanese green tea.	Animals had an inflammation induced by placing a silk thread ligature around maxillary molar for 7 days to accumulate plaque. Different concentrations (1.5 g, 3 g, and 6 g dried tea leaves per 60 mL of water) of Japanese green tea were used for watering the animals for 1–3 weeks after ligature removal. The tea infusion was obtained by putting the leaves for 90 s in a 70 °C water. Mice were sacrificed after the end of experiment. Histopathological analysis was performed, and Micro-CT were done for a vertical alveolar bone loss measurement.	Green tea significant inhibited ligature-induced bone loss. Moreover, it has alleviated the number of inflammatory cells and osteoclasts. The effect of green tea extract was found to be concentration and time dependent. The most significant therapeutic effect was obtained with 6 g/60 mL green tea at 1 week of administration.	Kaboosaya et al., 2020	[57]
Unverified commercial proanthocyanidin (PA) purchased at Sigma Chemical Co., St. Louis, MO, USA.	Male Wistar rats were divided into 3 groups: Group 1: Control Group 2: Rats with experimental periodontitis (EP), divided next to Group 3: 6 rats with experimental periodontitis (EP) received 30 mg PA (subcutaneously)/kg body weight for 30 days; and Group 4: 6 rats with experimental periodontitis (EP) received 20 mg tinidazole (administered orally)/kg body weight for 30 days.	Experimental periodontitis was induced by injecting *E. coli* endotoxin. After the treatment procedure, the animals were sacrificed and blood was collected to perform biochemical assays: hydrogen peroxide (H_2_O_2_), superoxide anion (O_2_^•−^), myeloperoxidase (MPO), lipid peroxides, fibrinogen assay, C-reactive protein (CRP), ascorbic acid, α-tocopherol, ceruloplasmin, reduced glutathione. Moreover, the animal maxilla halves were dissected, and they were analyzed using light microscopy.	Proanthocyanidins and tinidazole significant inhibition of reactive oxygen species and lipid peroxides. In group with PA (3) and tinidazole (4) significant decrease in the levels of acute phase proteins was observed compare to EP group (2). Moreover, PA (3) and tinidazole (4) significant increased levels of nonenzymatic antioxidants. Contrary to the EP group (2), no cellular infiltration of inflammatory cells was observed in the PA and tinidazole groups in histopathological examination.	Govindaraj et al., 2019	[51]
A commercial grape seed proanthocyanidin extract (GSPE) containing 50 mg polyphenols and 30 mg flavonoids in 100 mg	Animal study, 40 Wistar male rats were used: C group—Control group, P group—Periodontitis group, D group—Diabetes group, D+P group—Diabetes and periodontitis group, GSPE-100 group—Diabetes, periodontitis and 100 mg/kg/day GSPE group, GSPE-200 group—Diabetes, periodontitis and 200 mg/kg/day GSPE group.	GSPE were administered by oral gavage. The animals were sacrificed after 30 days. Alveolar bone loss was measured with a stereomicroscope. Level of (MMP)-8, VEGF and HIF-1alpha was defined with immunohistochemistry. Tartrate-resistant acid phosphatase-positive osteoclast cells were also determined. Total inflammatory cells (eosinophil, lymphocyte, neutrophil, and macrophage cells) in an area of 10,000 μm^2^ of periodontal ligament were counted.	The highest alveolar bone loss was observed in the group with induced diabetes + periodontitis (D+P) (*p* < 0.05). GSP-200 group significantly decreased alveolar bone loss (*p* < 0.05). In the D+P highest osteoclast count was found however the difference was not significant compared to the P, GSPE-100 and GSPE-200 groups. GSPE-100 and GSPE-200 groups significantly decreased inflammatory cell numbers compare to D+P group. The osteoblast numbers increased in the GSPE-100 and GSPE-200 groups compared to the P and D+P groups (*p* < 0.05). MMP-8 and HIF-1alpha levels were highest in the D+P group and GSPE significantly decreased these levels (*p* < 0.05).	Toker et al., 2018	[53]
Grape seed extract (GSE) was obtained from Berkem SA, (Gardonne, France), and supplied in a form of standardized extract containing >90% oligomeric proanthocyanidins	40 male Sprague Dawley rats. 4 equal groups were used. Group A was a positive control, the rats were fed only standard laboratory diet/water; Groups B, C, D experimental group rats that received GSE (same dose) for different periods of time. Group B—received GSE for two weeks before periodontitis induction and continued for six weeks; Group C—received GSE from the day of periodontitis induction and continued for six weeks. Group D—received GSE after ligature removal and continued for two weeks.	Animals had an inflammation induced by placing a 4-0 suture around mandibular molar. Sutures were kept for 4 weeks to induce periodontitis. GSE was systemically administered via gavage feeding every single a day in a dose of 200 mg/kg body weight. After animals were sacrificed, histological and immunohistochemical examination of the specimens were carried out.	The anti-inflammatory activity of the GSE was observed. Inflammatory cell number (ICN) was lower in the experimental groups with GSE. Clinical attachment level (CAL) was higher in experimental GSE groups. Osteoclast density (OD) was lower in experimental GSE groups compared to the control. In the gingival epithelium (GE) more IL-10 accumulation was determined in experimental groups compared to the control. No difference in TGF-β in GE was found between the groups.	Özden et al., 2017	[54]
Epigallocatechin-3-gallate (EGCG)	Animal study: 24 female mice were divided into 3 equal groups. The mice received distilled water: groups (1) and (2) distilled water or 0.02% solution of EGCG (group 3) from 8 weeks to 15weeks.	Mice were orally inoculated with (1) PBS or *Porphyromonas gingivalis* in PBS – groups (2) and (3). At the age of 15 weeks, the mice were euthanized to collect blood, gingival tissue and maxillae samples. Mouse inflammation antibody array C1 was used to detect the intensities of 40 mouse inflammatory mediators in serum. Cytokine levels were detected using ELISA kits for IL-17 and IL-1β in serum. The sections of gingival tissue were visualized with IL-17 and IL-1β specific immunostaining. The level of gene expression in the gingival tissue was determined using RT-qPCR. Alveolar bone resorption was analyzed by forming three dimensional structures using a microcomputed tomography.	EGCG significantly reduced *P. gingivalis* induced alveolar bone resorption. In serum sample, EGCG significantly decreased the high expressions (caused by *P. gingivalis* infection) of proteins such as: IL-1β, IL-6, IL-9 and IL-12p70, exotain-1, exotain-2, fas ligand, MCP-1, MIG, MIP-1α, whereas IL-17 and TNF-α were slightly decreased without being statistically significant. ELISA assay showed that EGCG reduced level of IL-17 and IL-1β in serum, however IL-17 level was not statistically significant. In the gingival tissue, EGCG reduced, increased by *P. gingivalis* infection, expression of IL-17 and IL-1β as well as significantly down-regulated the level of gene expression: IL-1β, IL-6, TNF-α, RANKL, CCL2 and MMP-9, but not IL-23. The expression of IL-17 and MMP-2 were slightly down-regulated but without statistically significant.	Cai et al., 2015	[55]
Non-dialyzable material (NDM) prepared from concentrated cranberry (*Vaccinium macrocarpon*) juice, rich in proanthocyanidins	Female BALB/c mice.	Mice (*n* = 16) were oral infected with *Porphyromonas gingivalis* and *Fusobacterium nucleatum* mixture (1:1). NDM (4 mg/mL) was added to the bacteria (in PBS) and the drinking water, whereas the control group received the infection in PBS alone. The maxillary jaws were harvested, and alveolar bone loss was evaluated by computed microtomography. Mice (*n* = 12) were challenged by an injection of PBS containing a mixture of *P. gingivalis* and *F. nucleatum* into the chambers of dorsolumbar area. In the experimental groups, NDM was added to the bacteria (in PBS) or to the PBS at a final concentration of 4 mg/mL, whereas the control group received the infection in PBS alone. Chamber exudates were harvested for analysis-TNF-α, quantified using two-site ELISA.	The NDM addition to the mixed infection reduced the alveolar bone loss induced by the mixed infection by approximately 20%. In subcutaneous chamber model of inflammation, the addition of NDM resulted in reduced of TNF-α levels, compared with group without NDM, at all tested times, however results were statistically significant only at 24 h post-infection, but not at 2 h.	Polak et al., 2013	[29]
Epigallocatechin-3-gallate (EGCG)	Male Sprague-Dawley rats divided into 2 groups: (1) Control group (*n* = 24; fed PBS vehicle after inducing experimental periodontitis), (2) Epigallocatechin-3-gallate group (*n* = 24; fed EGCG after inducing experimental periodontitis). All administration was conducted with an oral gavage on a daily basis.	Animals sacrificed 1, 2 and 4 weeks after EGCG or PBS administration. Histomorphometric and histologic analyses, tartrate resistant acid phosphatase staining and immunohistochemistry were carried out.	Administration of EGCG decreased the expression of IL-6 already in the early treatment period and decreased the expression of TNF at the 4th week of treatment. Downregulation of TNF and IL-6 expression by EGCG led to a reduction in the number and activity of osteoclasts, resulting in reduced bone loss. EGCG also reduced collagen destruction.	Cho et al., 2013	[56]
Commercial grape seed extract containing 95% proanthocyanidins (PA)	Male Wistar with experimentally induced periodontitis (by injecting *E. coli* endotoxin). The experimental group was divided into subgroups depending on the dose of subcutaneous proanthocyjanidin used (10–40 mg/kg body weight) and treatment time (10–30 days of treatment). One subgroup was treated with metronidazole (20 mg /kg body weight) for 30 days (administered orally).	After the experimental period, the rats were euthanized to collect blood, bone and teeth. Bone and teeth were obtained for the histopathological evaluation.	PA at an effective dose of 30 mg/kg body weight when administered subcutaneously, for 30 days caused a decrease in serum lipid peroxides, reactive oxygen species, lysosomal enzymes, acute phase proteins, and an increase in antioxidant levels. Histopathological evidence of experimental periodontitis showed cellular infiltration of inflammatory cells while PA treated groups demonstrated only scattered inflammatory cells.	Govindaraj et al., 2010	[52]
Epigallocatechin-3-gallate (EGCG)	20 Wistar rats divided into 2 equal groups: Animals were given intraperitoneal injections EGCG (80 mg/kg) or were given intraperitoneal injections of normal saline (NS, as control) on a daily basis until death. The animals were sacrificed after 20 days.	The jaws were dissected, and radiographs were taken. Cyr61 and CCL2 were measured using immunohistochemistry assays.	Administration of EGCG has significantly impaired a periapical bone resorption compared with the control. Moreover, the image analysis showed that EGCG suppressed periapical osteolysis by an average of 57.2%. EGCG diminished Cyr61 expression in osteoblast cells and, subsequently, macrophage chemotaxis into the lesions. A lower percentage of Cyr61-positive osteoblasts in the EGCG-treated group (21.3%), compared with that in the control group (62.1%) was observed.	Lee et al., 2009	[39]
Extract from the leaf of *Camelia sinensis,* containing 41.6% catechins	29 cats (mongrel; male 15, female 14) affected with gingivitis.	Cats were fed the commercial control diet for 14 days, prior to being put on the experimental diet for 45 days. The experimental diet with extract (0.4 mg/g or 0.8 mg/g) was prepared for the animals. Cats were fed twice daily during the test period. Gingival index (GI), oral malodor, and percentage of the genus *Porphyromonas* in the subgingival microbiota were examined.	Addition of catechin-rich extract resulted in a significant decrease in gingival index (more marked for 0.8 mg/g than 0.4 mg/g) and decrease the odor from the mouth, but significant only for diet with 0.8 mg/g extract. The experimental diet also caused a significant decrease in the percentage of *Porphyromonas sp.* (stronger for 0.8 mg/g than 0.4 mg/g).	Isogai et al., 2008	[58]

**Table 3:** Clinical studies.

Treatment	Study Design and Population	Methods	Results	Authors, Year	Ref.
The experimental treatment consisted of 90 mg supplement based on blueberry and red fruit rich in oligomeric proanthocyanidins (OPCs) (equivalent to 36 mg OPCs-oligomeric proanthocyanidins) and 120 mg of vitamin C	A prospective, double-blind, randomized, controlled clinical trial in the gingivitis prevention. 20 healthy volunteers took the experimental or placebo treatment for 21 days. The pill was maintained in the mouth until complete dissolution.	Two evaluation visits were performed—after 14 and 21 days. During clinical examination Silness and Löe index, the gingival bleeding index, the Turesky plaque index, the inflammatory crevicular fluid study (IL6), and changes in the brightness of the gingiva were evaluated.	The Silness and Löe gingival index was higher in the control group than in the experimental group. The bleeding was lower in the experimental group compared to the control group. In contrast to the above results, the amount of dental plaque was slightly higher (33%) in the experimental group versus in the control group. No significant differences between the study group and the control group were seen in brightness of the gingiva. Statistically significant differences in level of IL-6 were found at the baseline between the experimental group and the control group and in the subsequent visits. However, in experimental group level of IL-6 was lower.	Díaz Sánchez et al., 2017	[59]
Thermo-reversible sustained-release system incorporated with green tea extract	A controlled, split-mouth single-evaluator masked study was conducted to evaluate the effect of green tea extract as a sustained-release system in patients with chronic periodontitis (CP). 30 patients, each with 2 sites (test and control) having probing depths (PDs) of ≥4 mm, were selected. Green tea and placebo gels were placed at test and control sites as an adjunct to Phase 1 periodontal therapy.	Assessment of gingival index (GI), pocket depth (PD), and relative clinical attachment levels (rCALs) was done at baseline and at 4 weeks.	When the comparison of means of GI, PD, and rCAL was done between baseline values and at the end of 4 weeks within the test group, and control group all the parameters were lowered and statistically highly significant. The test group showed significantly better results when compared with controls. Adjunctive local drug therapy with thermo-reversible green tea gel has revealed to reduce pockets and inflammation during the 4 weeks of the clinical trial in patients with CP.	Chava and Vedula 2013	[60]
Hydroxypropylcellulose strips containing green tea catechin (Taiyo Kagaku, Mie, Yokkaichi, Japan)	6 volunteers with advanced periodontitis, but with no other systemic disorders. From each volunteer two pockets were selected: one for administration of the test agent and the other for placebo. Strips were applied in pockets in patients once a week for 8 weeks. The subjects were divided randomly into the scaled group (3 subjects) non-scaled group (3 subjects) were applied in pockets in patients.	The clinical PD, enzymatic (peptidase activities) and microbiological effects (the proportion of black-pigmented, Gram-negative anaerobic rods (BPR) of the catechin) were determined.	The PD and the BPR were decreased in the catechin group with mechanical treatment at week 8 compared to baseline. The peptidase activities in the gingival fluid were maintained at lower levels during the experimental period in the test sites with catechin, while it reached 70% of that at baseline in the placebo sites.	Hirsawa et al., 2002	[61]

## Data Availability

Not applicable.
